# Phylogeny, infrageneric classification and species delimitation in the Malagasy *Impatiens* (Balsaminaceae)

**DOI:** 10.3897/phytokeys.110.28216

**Published:** 2018-11-02

**Authors:** Elisette M. Rahelivololona, Eberhard Fischer, Steven B. Janssens, Sylvain G. Razafimandimbison

**Affiliations:** 1 Parc Botanique et Zoologique de Tsimbazaza, BP 4096, Antananarivo 101, Madagascar and Université de Mahajanga, Mahajanga I 401, Madagascar Université de Mahajanga Mahajanga Madagascar; 2 Institute for Integrated Sciences, Department of Biology, University of Koblenz-Landau, Universitätsstr. 1, D-56070 Koblenz, Germany University of Koblenz-Landau Koblenz Germany; 3 Meise Botanical Garden, Nieuwelaan 38, BE-1860 Meise, Belgium Meise Botanical Garden Meise Belgium; 4 Swedish Museum of Natural History, Department of Botany, Box 50007, SE-10405 Stockholm, Sweden Swedish Museum of Natural History Stockholm Sweden

**Keywords:** Balsaminaceae, infrageneric classification, *
Impatiens
*, Madagascar, monophyly, species delimitation, systematics, taxonomy

## Abstract

The species-rich genus *Impatiens* (Balsaminaceae) is represented in Madagascar by no less than 260 species. We conducted molecular phylogenetic analyses of the Malagasy *Impatiens* based on nuclear and plastid data and 52 accessions (representing 33 species) to: 1) reassess the monophyly of the Malagasy *Impatiens*; 2) assess the monophyly of the sections *Preimpatiens* (*Humblotianae* and *Vulgare* groups) with spurs and *Trimorphopetalum* without spurs as delimited by Perrier de la Bâthie, as well as that of the subgenera *Impatiens* and *Trimorphopetalum* as defined by Fischer and Rahelivololona; 3) infer the current status of some morphologically variable species; and 4) test whether the species of *Impatiens* from the Marojejy National Park form a monophyletic group. The Malagasy *Impatiens* are further confirmed to be paraphyletic with respect of the Comorian *I.auricoma.* The present sectional and subgeneric classifications of the Malagasy *Impatiens* are partly supported, with strong support for the monophyly of the sect. or subgen. Trimorphopetalum. Section Preimpatiens was not supported as monophyletic and neither the *Humblotianae* group nor the *Vulgare* group is monophyletic. *Impatienselatostemmoides*, *I.* “*hammarbyoides*”, *I.inaperta*, *I.lyallii* and *I.manaharensis* are either para- or polyphyletic and may represent morpho-species. The *Impatiens* species from the Marojejy National Park do not form a monophyletic group and therefore are suggested to be derived from numerous independent colonisation events from all over Madagascar followed by subsequent diversifications.

## Introduction

**Figure 1.**
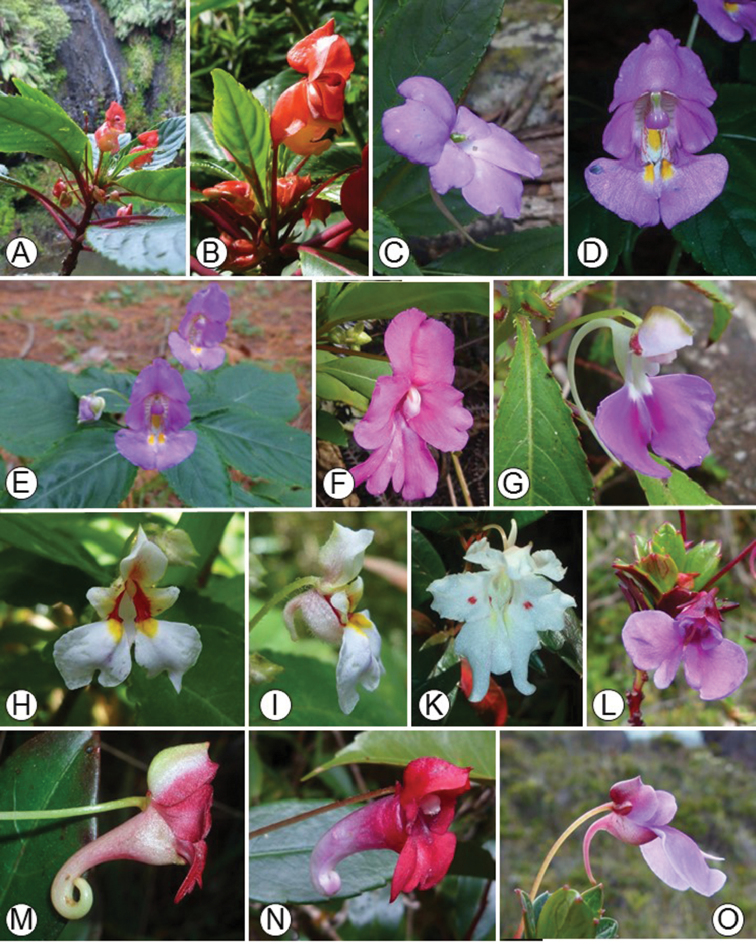
Representatives of Impatienssubgen.Impatiens. **A, B***Impatiensbicaudata*, Montagne d’Ambre **C***I.lyallii*, Montagne d’Ambre **D, E***I.bisaccata*, Montagne d’Ambre **F***I.max-huberi*, Marojejy **G***I.nomenyae*, Marojejy **H, I***I.masoalensis*, Marojejy **K**I.cf.manaharensis, Marojejy **L, O***I.marojejyensis*, Marojejy **M***I.susan-nathansoniae*, Marojejy **N***I.hendrikii*, Marojejy. Photos: E. Fischer.

**Figure 2.**
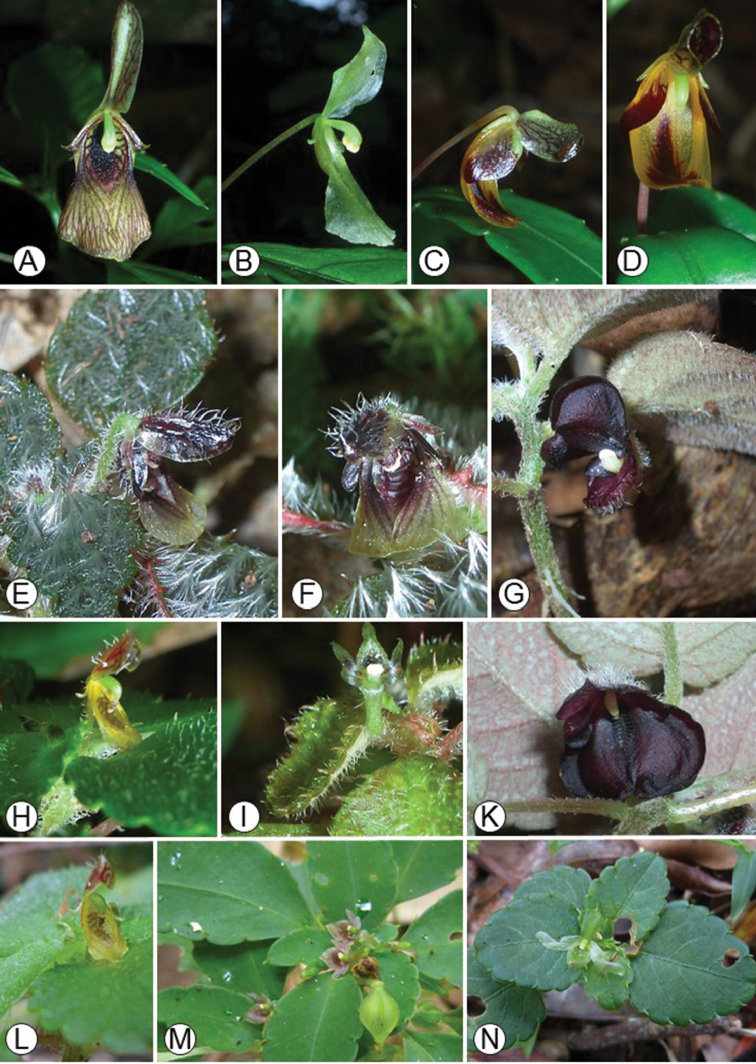
Representatives of Impatienssubgen.Trimorphopetalum. **A***Impatienslutzii*, Montagne d’Ambre **B***I.galactica*, Marojejy **C, D***I.* “*capuronii*”, Marojejy **E, F***I.furcata*, Marojejy **G, K***I.navicula*, Marojejy **H, L***I.* “*humillima*”, Marojejy **I***I.* “*hammarbyoides*”, Marojejy **M***I.elatostemmoides*, Marojejy **N**I.sp. nov.aff.elatostemmoides 3, Montagne d’Ambre. Photos: E. Fischer.

The genus *Impatiens* L. (Balsaminaceae) is a monophyletic and diverse genus with more than 1000 species (e.g. Yuan et al. 2004, Janssens et al. 2009) and is represented by at least 260 endemic species in Madagascar (e.g. Fischer and Rahelivololona 2002, 2004a, b, 2015a, b, c, 2016, Fischer et al. 2003, 2017). This continental island is one of the centres of species diversity for the genus, which is the largest flowering plant genus on the island (Perrier de la Bâthie 1934, 1948, Humbert 1955, 1956, Fischer and Rahelivololona 2002, 2004a, b, 2007a, b, 2015a, b, c, 2016, Fischer et al. 2003, 2017) (Figs 1, 2). Fischer and Rahelivololona (e.g. 2002, 2004a, b) initiated the taxonomic studies of the Malagasy and Comorian members of *Impatiens* in an attempt to produce an updated Flora of the family Balsaminaceae for Madagascar and the Comoros. Since then, 75 new species have been described and at least another 75 new species will be published in the near future (E. Rahelivololona and E. Fischer, unpubl. data). The majority of the Malagasy *Impatiens* occurs in the montane regions of northern and eastern Madagascar (e.g. Tsaratanana National Park with 36 species, Marojejy National Park with 48 species, Masoala National Park with 59 species) (Figs 1, 2).

Warburg and Reiche (1895) provided the first global infrageneric classification for *Impatiens* based solely on morphological data. Since then, a number of infrageneric classifications of the genus have been proposed for some tropical regions (including Madagascar). Perrier de la Bâthie (1934) established the first sectional classifications for the Malagasy and Comorian *Impatiens*, placing the species with obvious spurs and anthers with apical dehiscence in his section Preimpatiens. The author subdivided sect. Preimpatiens into two groups: the *Vulgare* group with pink, purple, white or orange flowers with shorter and slender spurs and the *Humblotianae* group with red, yellow or orange flowers with larger and broader spurs. Furthermore, Perrier de la Bâthie (1934) classified the Malagasy *Impatiens* species with anthers dehiscing apically but without spurs on the low sepals into two sections: the monotypic sect. Impatientella with deltoid and sharp anthers and sect. Trimorphopetalum with obtuse or truncate anthers (Fig. 2), both endemic to Madagascar. Fischer and Rahelivololona (2002) recognised Perrier de la Bâthie’s sect. Impatiens with spurs (Fig. 1) and sect. Trimorphopetalum without spurs (Fig. 4) at subgeneric level. They formally subsumed sect. Impatientella into subgen. Trimorphopetalum.

The category of species is widely accepted as the basic or working unit of biological classification (Rosell et al. 2010, Hohenegger 2014). From a phylogenetic point of view, a species of traditional taxonomy is often viewed as a species hypothesis tested by recovering either monophyletic or non-monophyletic units. Most species concepts (e.g. Baum and Shaw 1995) consider monophyly to be congruent with species hypotheses, while almost all species concepts view polyphyly as a rejection of species hypotheses (e.g. Rosell et al. 2010). Previous and contemporary workers (e.g. Perrier de la Bâthie 1934, Fischer and Rahelivololona 2002, Fischer et al. 2003, 2017), dealing with species circumscription in the Malagasy *Impatiens*, have encountered difficulties in deciding whether a taxon represents a “real” species or a morpho-species. Several species (e.g. *I.elatostemmoides* H.Perrier, *I.* “*hammarbyoides*” Eb.Fisch. & Raheliv. (nomen provisorium, not yet published), *I.inaperta* (H.Perrier.) H.Perrier, *I.lyallii* H.Perrier and *I.manaharensis* Baill.) are known to be morphologically variable and this raises doubts as to whether these species deserve specific status. Molecular trees (phylogenetic hypotheses) can identify mono-, para- or polyphyletic taxa and can therefore be an important tool for assessing species delimitation. In addition, phylogenetic trees can also be used for assessing the various infrageneric classifications of the Malagasy *Impatiens*.

The first molecular phylogenetic study of the family Balsaminaceae by Yuan et al. (2004), based on nuclear ribosomal ITS (nrITS) sequence data and including 18 Malagasy *Impatiens* species (six species from subgen. Trimorphopetalum and 12 species from subgen. Impatiens), indicated that the Malagasy *Impatiens* species were polyphyletic, as they were resolved into three groups: a *Humblotianae*-*Vulgare*-*Trimorphopetalum* clade, a lineage with *I.baroni* Baker of sect. Impatiens and a *Humblotianae*-*Vulgare* clade consisting of seven Malagasy species of sect. Impatiens (*I.anovensis* H.Perrier to *I.vilersii* Costantin & Poiss.). The authors also showed the monophyly of the Malagasy sect. Trimorphopetalum only if sect. Impatientella, containing the spurless and entirely cleistogamous species *I.inaperta*, is included. Their results suggested a Malagasy origin of the Comorian species. In addition, each of the Malagasy *Impatiens* clades was nested within an African *Impatiens* lineage, suggesting multiple African origins of the Malagasy *Impatiens*. Moreover, subgen. Impatiens was not monophyletic. In contrast to Yuan et al. (2004), Janssens et al. (2006, 2007, 2009), who included representatives of subgen. Impatiens and *Trimorphopetalum*, strongly supported the monophyly of the Malagasy *Impatiens* (including the Comorian *I.auricoma*). These last three studies also confirmed a single African origin of the Malagasy representatives. However, all of the above-mentioned molecular studies (Yuan et al. 2004 with 17 species; Janssens et al. 2006, 2007 and 2009 with six species) used a very limited sampling of the Malagasy *Impatiens* and, therefore, the monophyly of subgen. Trimorphopetalum was in need of being tested with a much larger sampling effort. More recently, based on both morphological and molecular evidence, Yu et al. (2015) divided the genus Impatiens into two subgenera, subgen. Clavicarpa and subgen. Impatiens. Moreover, the authors delineated seven sections in subgenus Impatiens: sect. Semeiocardium, sect. Tuberosae, sect. Racemosae, sect. Impatiens, sect. Scorpioidae, sect. Fasciculatae and sect. Uniflorae. Of these, the latter is characterised by short fusiform capsules and includes all Malagasy species of *Impatiens*, as well as several African and Asian species.

**Figure 3.**
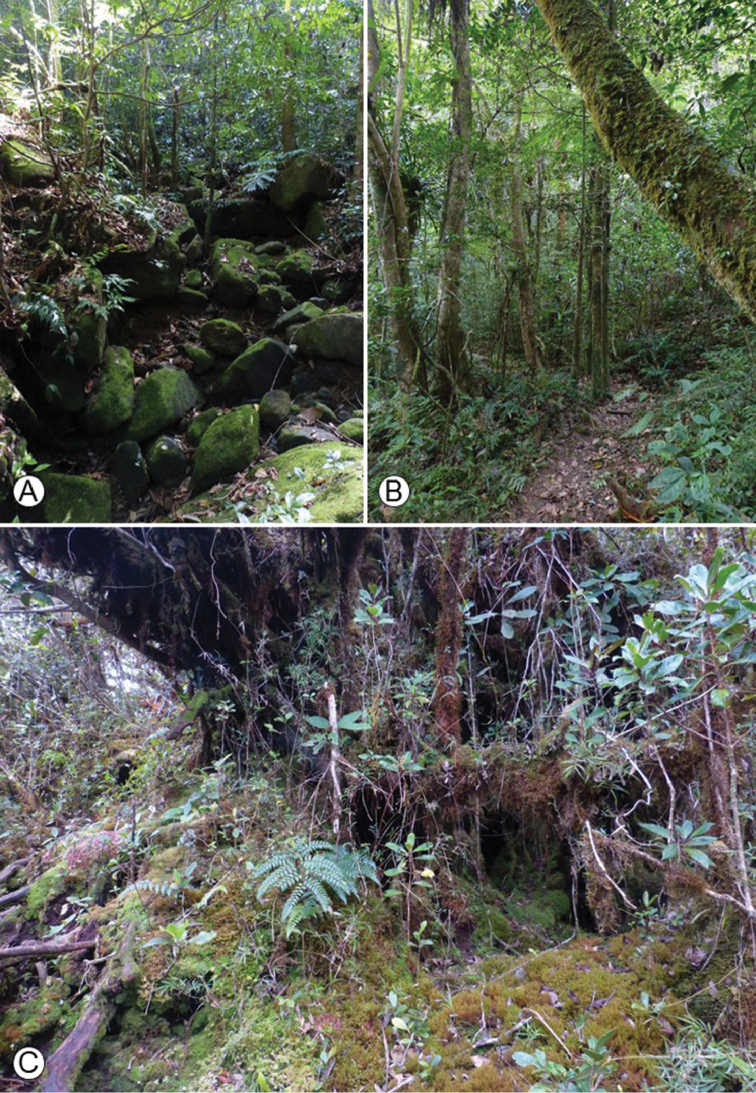
**A, B** Lowland rainforest of the Marojejy National park **A** ca. 400 m **B** ca. 490 m **C** Moist montane rainforest of the Marojejy National park at ca. 1100 m. Photo: E. Fischer.

**Figure 4.**
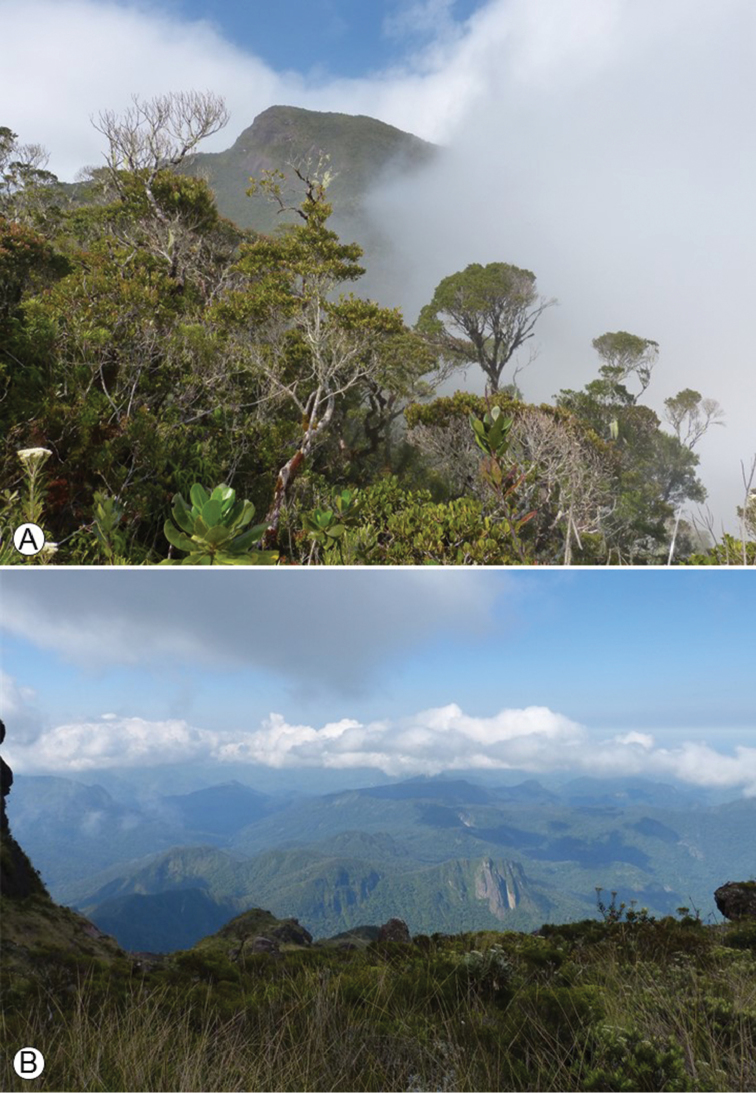
**A** Sclerophyllous montane cloud forest of the Marojejy National park; 1500 m **B** Subalpine grassland, Marojejy at ca. 2100 m. Photo: E. Fischer.

The Marojejy National Park is located in north-eastern Madagascar within the SAVA Region. With its tallest peak rising to 2137 m, the area is home to a diverse flora of upland species. The wide range of elevations and rugged topography of Marojejy create diverse habitats, which transition quickly with changes in altitude. There are four types of forests within the park: lowland rainforest below 800 m (Fig. 3); moist montane rainforest between 800 and 1400 m (Fig. 3); sclerophyllous montane cloud forest between 1400 and 1800 m; and ericoid shrub above 1800 m (Fig. 4) (Humbert 1955). The higher summits are covered by subalpine grassland with small ericaceous shrubs (Fig. 4), and are home to numerous local endemic species of *Impatiens* and of other large genera, such as *Streptocarpus* (Gesneriaceae) and *Helichrysum* (Asteraceae). The park has been recognised as a marked centre of plant endemism. For example, 32 palm species found in the Marojejy area are endemic to Madagascar and seven of them are restricted to the park. Of the 18 species of tree ferns, inventoried in the rainforests of Marojejy, seven are endemic to the area (Madagascar Catalogue 2017). Whether the endemic species of *Impatiens* from the Marojejy form a monophyletic group or are the result of a mixture of colonisation events from other regions through time have yet to be assessed.

The main objective of this study was to reconstruct a new and larger phylogeny of the Malagasy *Impatiens*, with a particular emphasis on taxa collected from Marojejy, using two nuclear *AP3*/*DEF* homologues (*ImpDEF1* and *ImpDEF2*) and the plastid *atpB-rbcL* spacer. The resulting phylogeny was subsequently used to (i) reassess the monophyly of the Malagasy *Impatiens* as stated by Janssens et al. (2006, 2007, 2009); (ii) assess the monophyly of the sections *Preimpatiens* (Humblotianae and Vulgare groups) and *Trimorphopetalum* as delimited by Perrier de la Bâthie (1934), as well as that of the subgenera *Impatiens* and *Trimorphopetalum* (including sect. Impatientella) as defined by Fischer and Rahelivololona (2002); (iii) assess the current species status of the morphologically variable species *I.elatostemmoides*, *I.* “*hammarbyoides*”, *I.inaperta* and *I.manaharensis*, using monophyly as the primary criterion (Backlund and Bremer 1998); and iv) test whether the species of *Impatiens* from the Marojejy National Park form a monophyletic group. The sectional classification proposed by Yu et al. (2015) could not be assessed, as our sampling was solely addressing the Malagasy and Comorian *Impatiens* species.

## Methods

### Taxon sampling

The taxon sampling for this study was focused on the Malagasy representatives of the genus *Impatiens*. We expanded the previous dataset of Yuan et al. (2004) and Janssens et al. (2009) with 29 new accessions (Table 1). In total, 52 accessions were included in our analyses, representing 48 Malagasy specimens (representing about 31–33 species), two Comorian accessions (representing one species) and two African species (Table 1). This sampling represented the three major groups defined by Perrier de la Bâthie (1934) as occurring in Madagascar and the Comoros: 31 accessions from sect. Preimpatiens (Perrier de la Bâthie, 1934) or subgen. Impatiens (Fischer & Rahelivelolona, 2002) (10 accessions representing six species of the *Humblotianae* group; 21 accessions representing 11or 12 species of the *Vulgare* group); and 22 accessions representing about 14 species from sect. Trimorphopetalum (Perrier de la Bâthie, 1934) or subgen. Trimorphopetalum (Fischer & Rahelivololona, 2002). The species showing some morphological variation were represented by more than one individual and, thus, were the subject of a test for monophyly. Twenty-seven accessions, represented by at least 17 species, were from the Marojejy National Park. *Impatienscecilii* and *I.hydrogetonoides*, both from Africa, were used as outgroup based on Janssens et al. (2006, 2009).

**Table 1.** List of taxa studied, voucher information and accession numbers of the selected markers. ‘–’ refers to a missing sequence.

Taxa	Voucher information	nrITS	*atp*B–*rbc*L	ImpDEF1	ImpDEF2
*Impatiensandringitrensis* H.Perrier	Bot. Gard. Bonn 36655 (BONN), Madagascar	–	MH157104	MH157123	–
*Impatiensauricoma* Baill. 1	Bot. Gard. Bonn 34154 (BONN), Comores	–	DQ147815	EF133562	EF133615
*Impatiensauricoma* Baill. 2	E. Fischer 1270 (Bot. Gard. Zürich, E.Fischer s.n.) (BONN), Comores	MH881113	MH881068	–	–
*Impatiensbicaudata* H.Perrier 1	E. Fischer 1340 (Bot. Gard. Bonn 36586) (BONN), Madagascar	MH881114	MH881069	–	MH881160
*Impatiensbicaudata* H.Perrier 2	E. Fischer 1437 (BONN), Madagascar	MH881115	MH881070	MH881199	MH881161
*Impatiensbicaudata* H.Perrier 3	Bot. Gard. Zürich, E.Fischer s.n. (BONN), Madagascar	–	–	MH881198	–
*Impatiensbisaccata* H.Perrier 1	E. Fischer 1271 (Bot. Gard. Bonn 36496) (BONN), Madagascar	MH157152	MH881071	–	–
*Impatiensbisaccata* H.Perrier 2	E. Fischer 1435 (BONN), Madagascar	MH881117	MH881072	MH881200	–
*Impatiens* “*capuronii*” Humb. ex Eb.Fisch. & Raheliv. ined.	E. Fischer 1432 (Bot. Gard. Bonn 36427) (BONN), Madagascar	MH157171	MH157106	MH157127	MH157135
*Impatienscatati* H.Perrier 1	E. Fischer 1347 (Bot. Gard. Bonn 35920) (BONN), Madagascar	MH881120	MH881074	–	–
*Impatienscatati* H.Perrier 2	E. Fischer 1278 (Bot. Gard. Bonn 28424) (BONN), Madagascar	MH157142	FJ826634	FJ826686	–
*Impatienscecili* N.E.Br.	Knox 4353 (LV), Zimbabwe	–	FJ826635	FJ826687	FJ826741
Impatienscf.manaharensis Baill.2	E. Fischer 1427 (BONN), Madagascar	MH881123	MH881078	MH881204	MH881166
Impatienscf.manaharensis Baill. 3	E. Fischer 1348 (Bot. Gard. Bonn 36384)(BONN), Madagascar	MH881139	–	–	MH881182
*Impatienselatostemmoides* H.Perrier 1	E. Fischer 1284 (Bot. Gard. Bonn 26821) (BONN), Madagascar	MH157156	MF567403	–	MF567460
*Impatienselatostemmoides* H.Perrier 2	E. Fischer 1420 (BONN), Madagascar	MH881124	MH881080	MH881205	–
Impatienssp. nov.aff.elatostemmoides H.Perrier 3	E. Fischer 1439 (BONN), Madagascar	MH881110	MH881065	MH881194	MH881156
*Impatienselatostemmoides* H.Perrier 4	E. Fischer 1429 (BONN), Madagascar	MH881121	MH881076	–	–
*Impatienselianae* S.Abrahamczyk & Eb.Fisch	E. Fischer 1326 (Bot. Gard. Bonn 36144) (BONN), Madagascar	MH157157	MF567404	–	MF567461
*Impatienseriosperma* H.Perrier	E.Fischer 1342 (Bot. Gard. Bonn 35921) (BONN), Madagascar	MH157158	MF567414	–	MF567466
*Impatiensfurcata* H.Perrier	E. Fischer 1441 (BONN), Madagascar	MH881127	MH881083	MH881206	MH881170
*Impatiensgalactica* Eb.Fisch., Raheliv. & S.Abrahamczyk 1	E. Fischer 1319 (Bot. Gard. Bonn 36393) (BONN), Madagascar	MH881153	MH881107	–	–
*Impatiensgalactica* Eb.Fisch., Raheliv. & S.Abrahamczyk 2	E. Fischer 1426 (BONN), Madagascar	MH881128	MH881108	MH881225	MH881192
*Impatiens* „*hammarbyoides*“ Eb.Fisch. & Raheliv. 1 ined.	E. Fischer 1430 (BONN), Madagascar	MH157165	MF567417	MF567445	MF567469
*Impatiens* “*hammarbyoides*” Eb.Fisch. & Raheliv.2 ined.	E. Fischer 1447 (Bot. Gard. Bonn 37437) (BONN), Madagascar	MH157144	MH157099	MH157121	–
*Impatienshendrikii* Eb.Fisch. &Raheliv. 1	E. Fischer 1445 (BONN), Madagascar	MH881130	MH881086	MH881209	MH881173
*Impatienshendrikii* Eb.Fisch. &Raheliv. 2	E. Fischer 1440 (BONN), Madagascar	MH881129	MH881085	MH881208	MH881172
*Impatiens* “*humillima*” Humb. Eb.F isch. & Raheliv. ined.	E. Fischer 1431 (BONN), Madagascar	MH881131	MH881087	MH881210	MH881174
*Impatienshydrogetonoides* Launert	Dessein 719 (BR), Zambia	–	FJ826648	FJ826699	FJ826755
*Impatiensinaperta* (H-Perr.) H.Perrier 1	E. Fischer 1346 (Bot. Gard. Bonn 27467) (BONN), Madagascar	–	MH157109	–	–
*Impatiensinaperta* (H-Perr.) H.Perrier 2	E. Fischer 1357 (BONN), Madagascar	MH881132	MH881089	–	–
*Impatiensinaperta* (H-Perr.) H.Perrier 3	E. Fischer 1448 (BONN), Madagascar	MH881133	MH881090	MH881213	MH881177
*Impatienslaurentii* Eb.Fisch. & Raheliv.	E. Fischer 1293 (Bot. Gard. Bonn 36132) (BONN), Madagascar	MH157159	–	MH157120	–
*Impatienslutzii* Eb.Fisch. & Raheliv. 1	E. Fischer 1318 (Bot. Gard. Bonn 36381) (BONN), Madagascar	MH881135	MH881092	–	MH881179
*Impatienslutzii* Eb.Fisch. & Raheliv. 2	E. Fischer 1438 (BONN), Madagascar	MH881136	MH881093	MH881214	MH881180
*Impatienslyallii* H.Perrier 1	E. Fischer 1294 (Bot. Gard. Bonn 152a) (BONN), Madagascar	MH157169	MF567420	MF567448	MF567471
*Impatienslyallii* H.Perrier 2	E. Fischer 1341 (Bot. Gard. Bonn 152b)(BONN), Madagascar	MH881138	MH881094	–	–
*Impatiensmanaharensis* Baill. 1	E. Fischer 1434 (Bot. Gard. Bonn 36384) (BONN), Madagascar	MH881139	MH881077	MH881203	MH881182
*Impatiensmandrakae* Eb.Fisch. & Raheliv.	E. Fischer 1345 (Bot. Gard. Bonn 26822) (BONN), Madagascar	MH157166	MF567421	–	MF567472
*Impatiensmarojejyensis* Humbert & H.Perrier	E. Fischer 1444 (BONN), Madagascar	MH881141	MH881096	MH881215	MH881184
*Impatiensmasoalensis* H.Perrier 1	E. Fischer 1443 (BONN), Madagascar	MH881143	–	MH881216	–
*Impatiensmasoalensis* H.Perrier 2	E. Fischer 1424 (Bot. Gard. Bonn 36386) (BONN), Madagascar	MH157161	MF567422	MF567449	MF567473
*Impatiensmasoalensis* H.Perrier 3	E. Fischer 1424 (BONN), Madagascar	MH881144	–	MH881217	MH881186
*Impatiensmax-huberi* Eb.Fisch. &Raheliv.	E. Fischer 1421 (Bot. Gard. Bonn 36428) (BONN), Madagascar	MH157147	MH157110	MH157116	MH157137
I*mpatiens navicula* Eb.Fisch. & Raheliv. 1	E. Fischer 1422 (BONN), Madagascar	MH881147	MH881101	MH881220	MH881189
*Impatiensnavicula* Eb.Fisch. & Raheliv. 2	E. Fischer 1446 (BONN), Madagascar	MH881146	MH881100	MH881219	MH881188
*Impatiensnomenyae* Eb.Fisch. & Raheliv.	E. Fischer 1425 (BONN), Madagascar	MH881148	MH881102	MH881221	–
*Impatiensrenae* Eb.Fisch. & Raheliv.	E. Fischer 1442 (BONN), Madagascar	MH881149	MH881103	MH881222	–
*Impatiensrutenbergii* O.Hoffm	E. Fischer 1310 (Bot. Gard. Bonn 37463) (BONN), Madagascar	MH881150	MH881104	–	MH881190
*Impatiensscripta* H.Perrier	E. Fischer 1423 (BONN), Madagascar	MH881151	MH881105	MH881223	MH881191
Impatiens sp. nov. aff. lyallii	E. Fischer 1428 (BONN), Madagascar	MH881152	MH881106	MH881224	–
*Impatienssusan-nathansoniae* Eb.Fisch. & Raheliv.	E. Fischer 1433 (BONN), Madagascar	MH881155	MH881109	MH881226	MH881193

### Molecular protocols

Total genomic DNA was isolated from silica-dried leaf material using a modified CTAB protocol (Doyle and Doyle 1987), which was optimised for *Impatiens* by Janssens et al. (2006, 2009). The two nuclear *AP3*/*DEF* homologues (*ImpDEF1* and *ImpDEF2*) and the plastid *atpB-rbcL* intergenic spacer were amplified following Janssens et al. (2007) and Janssens et al. (2006). PCR reactions for all three gene markers investigated in this study consisted of 2 min initial denaturation at 94 °C and 30 cycles of 30 s denaturation at 94 °C, 30 s primer annealing at primer specific temperature and 1 min extension at 72 °C. Primer annealing for *ImpDEF1*, *ImpDEF2* and *atpB-rbcL* were at 57 °C, 55.5 °C and 51 °C, respectively. Amplification reactions were carried out on a Gene Amp PCR system 9700 (Applied Biosystems). Purified amplification products were sent to Macrogen, Inc. (Seoul, South Korea) for sequencing. Sequences obtained in this study will be deposited at GenBank (Table 1).

### Data analyses

Contiguous sequences were assembled using Geneious v7.0.6 (Biomatters, New Zealand). Automatic alignment of the datasets was carried out with MAFFT (Katoh et al. 2002) under an E-INS-i algorithm, a 100PAM/k=2 scoring matrix, a gap open penalty of 1.3 and an offset value of 0.123. Subsequent manual fine-tuning of the aligned dataset was done in Geneious v7.0.6. Congruency between the nuclear and chloroplast datasets was inferred by a partition homogeneity test as implemented in PAUP*4.0b10a (Swofford 2000). The best-fit nucleotide substitution model for each plastid and nuclear dataset was determined using jModel Test 2.1.4 (Posada 2008) under the Akaike information criterion (AIC). The GTR+I+G model was found as best fit for *ImpDEF1*, whereas the GTR+G model was calculated as best substitution model for *ImpDEF2* and *atpB-rbcL*. A mixed-model approach was used in which the combined dataset was partitioned in order to apply a different model of evolution on each DNA region (Ronquist and Huelsenbeck 2003). Bayesian Inference (BI) analyses were conducted with MrBayes v3.1 (Huelsenbeck and Ronquist 2001) on three individual data partitions and a combined data matrix. Each analysis ran two times for 10 million generations. Trees were sampled every 2500 generations. Inspection of chain convergence and ESS parameters was done with TRACER v1.4 (Rambaut and Drummond 2007). Bayesian posterior probability (BPP) values between 0.50 and 0.95 were considered to be weakly supported, whereas BPP values above or equal to 0.95 were taken into consideration to indicate well-supported branches (Suzuki et al. 2002, Alfaro et al. 2003). Maximum Likelihood analyses were carried out on the CIPRES web portal using RAxML v7.2.8 (Stamatakis et al. 2008) under the GTRGAMMA model. Non-parametric ML bootstrapping analysis was calculated with 1000 bootstrap replicates.

## Results

**Figure 5.**
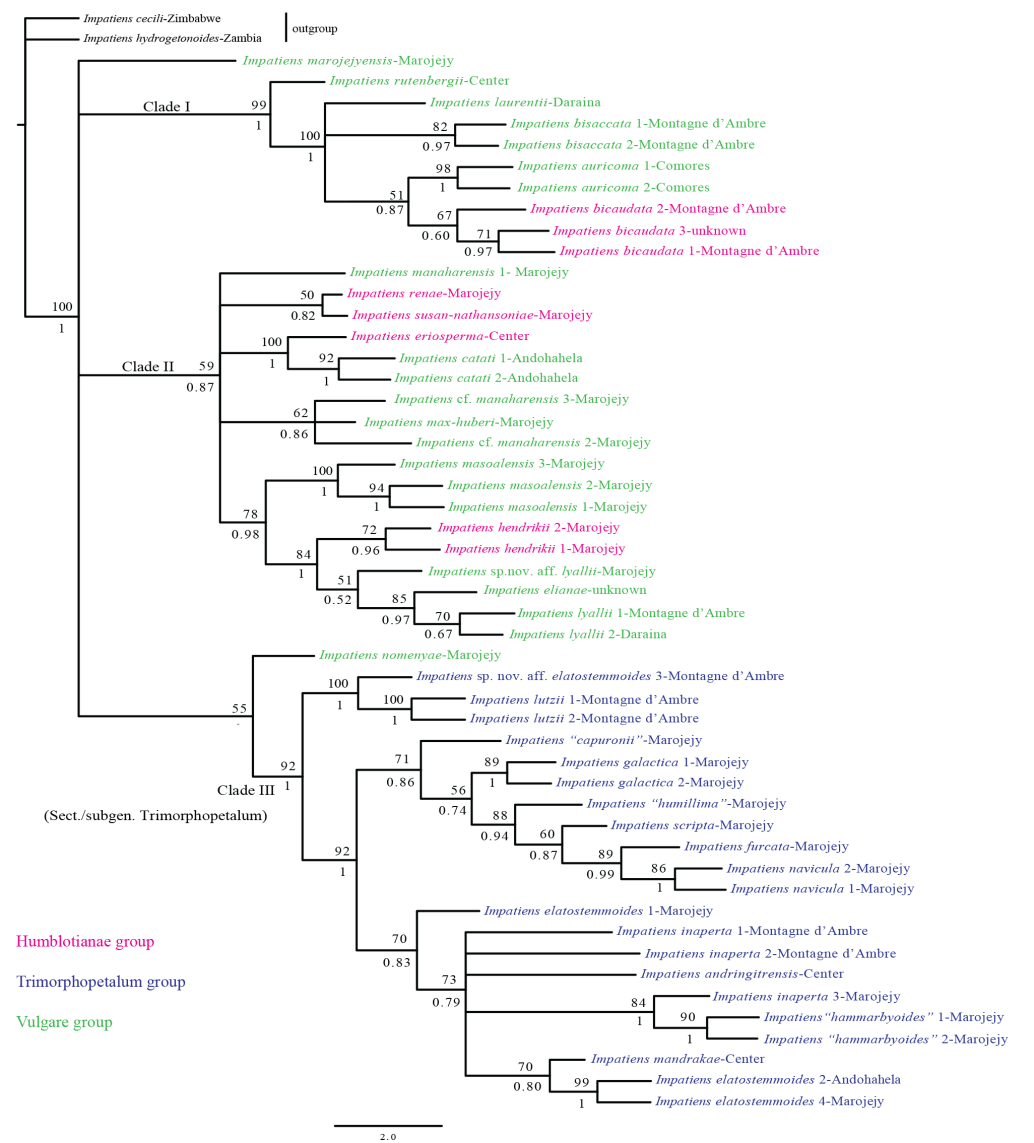
Maximum likelihood tree based on the combined nuclear-plastid data. Bootstrap support values and Bayesian posterior probabilities are above and below nodes, respectively.

The aligned *atpB-rbcL* and *ImpDEF1*/*ImpDEF2* matrices contained 924 bp and 812 bp, respectively. The phylogenetic tree, based on the combined data, is shown in Figure 5. The monophyly of the Malagasy *Impatiens* was not supported by our analyses, as the Comorian species *I.auricoma* was deeply nested within the Malagasy *Impatiens* Clade I (BS: 99; BPP: 1). Therefore, the Malagasy *Impatiens* are paraphyletic, unless the Comorian Impatiens species are included. The sect. Preimpatiens sensu Perrier de la Bâthie (1934)/subgen. Impatiens sensu Fischer and Rahelivololona (2002) was not resolved as a monophyletic group. Neither the *Humbotianae* group nor the *Vulgare* group was supported as monophyletic (Fig. 5). However, sect. Trimorphopetalum sensu Perrier de la Bâthie (1934)/subgen. Trimorphopetalum sensu Fischer and Rahelivololona (2002) was strongly supported as a monophyletic group (BS: 92; BPP: 1). The earliest diversified lineages in the Malagasy *Impatiens* clade fell into a large polytomy containing five groups (Fig. 5): *I.marojejyensis* Humbert & H.Perrier (member of the *Vulgare* group), *Impatiens* Clade I (formed by the representatives of the *Humblotianae* and *Vulgare* groups) (BS: 99; BPP: 1), *Impatiens* Clade II (also formed by the representatives of the *Humblotianae* and *Vulgare* groups) (BS: 59; BPP: 0.87), *I.nomenyae* Ed.Fisch. & Raheliv. and a strongly supported Clade III (BS: 92; BPP: 1) (formed by the representatives of the *Trimorphopetalum*). The phylogenetic relationships amongst these major lineages were unresolved. Within the *Impatiens* Clade II, the morphologically variable *I.manaharensis* was not supported as monophyletic. Within the *Impatiens* Clade III, the morphologically variable species *I.elatostemmoides* appeared polyphyletic, while *I.* “*hammarbyoides*”, *I.lyallii* and *I.inaperta*, also variable, seemed paraphyletic. Finally, the sampled species of *Impatiens* from the Marojejy National Park did not form a monophyletic group, as they were scattered across the tree (Fig. 5).

## Discussion

The present analyses confirm the paraphyly of the Malagasy *Impatiens* with respect to the Comorian *I.auricoma*. This is consistent with Janssens et al. (2006, 2007, and 2009) but inconsistent with the polyphyly of the Malagasy *Impatiens* as shown by Yuan et al. (2004). The monophyly of sect. Trimorphopetalum (*I.inaperta* included) suggested by Yuan et al. (2004) is further strongly supported (BS: 92; BPP: 100) by the present study. In other words, subgen. Trimorphopetalum, as delimited by Fischer and Rahelivololona (2002), is supported. Spurless, greenish, brown to blackish or yellowish (never white, pink or purple) flowers with boat-shaped lower sepals, obtuse or truncate and apically dehiscing anthers and the lack of extrafloral nectaries on leaf lamina and petioles are the synapomorphic characters for this lineage, which seems to have evolved from a common ancestor with spurs (Fig. 5). In Yuan et al. (2004), the *Trimorphopetalum* clade was resolved as the most derived within *Impatiens*. The authors argued that this spurless lineage could not be recognised at sectional or subgeneric level, as proposed by Perrier de la Bâthie (1934) and Fischer and Rahelivololona (2002), respectively, because this taxonomic decision seems to make sect. Preimpatiens sensu Perrier de la Bâthie (1934) or I.subgen.Impatiens sensu Fischer and Rahelivololona (2002) paraphyletic. Our results do not support or reject Yuan et al. (2004)’s claims, as the Malagasy *Impatiens* clade (including the Comorian *I.auricoma*) is largely unresolved (Fig. 5). Neither the sampled species from the *Vulgare* group nor those from the *Humblotianae* group form a monophyletic group, a result consistent with Yuan et al. (2004). Therefore, our results provide no support for the *Vulgare* group characterised by shorter and slender spurs or for the *Humblotianae* group defined by larger and broader spurs, as delimited by Perrier de la Bâthie (1934). Furthermore, our analyses do not support or reject the monophyly of sect. Preimpatiens sensu Perrier de la Bâthie (1934) or subgen. Impatiens sensu Fischer and Rahelivololona (2002). To summarise, this study partly supports the sectional and subgeneric classifications of the Malagasy *Impatiens* proposed by Perrier de la Bâthie (1934) and Fischer and Rahelivololona (2002). More molecular data are needed to further assess the monophyly of sect. Preimpatiens or subgen. Impatiens.

In addition, results of this molecular phylogenetic study further highlight the difficulties that the *Impatiens* taxonomists have faced when dealing with the species delimitation of the Malagasy *Impatiens* (e.g. Perrier de la Bâthie 1934; Humbert 1956; Fischer and Rahelivololona 2002, 2004a, 2007, 2015a, b, c). *Impatiensmanaharensis* seems polyphyletic and this supports our suspicion in the field that at least two taxa with very different morphology could be distinguished within this variable species. *Impatienselatostemmoides* seems polyphyletic, while *I.inaperta, I.* “*hammarbyoides*” and *I.lyallii* (Fig. 5) appear paraphyletic. Therefore, these morphologically variable species may well represent morpho-species, meaning that they represent a group of several different species or are parts of a species complex. As a consequence, this study indicates that the current species delimitation of these para- or polyphyletic species is in need of revision.

Finally, the *Impatiens* taxa from the Marojejy National Park do not form a monophyletic group, as they are spread across the tree (Fig. 5). This suggests that they are the result of numerous independent colonisation events from elsewhere in Madagascar, followed by subsequent diversifications. In other words, they seem to have had multiple origins.

## Conclusions

The Malagasy *Impatiens* are paraphyletic with respect of the Comorian *I.auricoma.* The present subgeneric and sectional classifications of the Malagasy *Impatiens* are partly supported, with strong support for the monophyly of subgen. Trimorphopetalum. Neither the *Humblotianae* group nor the *Vulgare* group forms a monophyletic group. *Impatienselatostemmoides*, *I.* “*hammarbyoides*”, *I.inaperta* and *I.manaharensis* are either para- or polyphyletic and may represent morpho-species. The *Impatiens* species from Marojejy do not form a natural group. A further study based on a much larger molecular data set and sampling from the entire geographic ranges of *Impatiens* in Madagascar is needed to produce a well-resolved phylogeny. This will hopefully allow for a retest of the monophyly of sect. Preimpatiens sensu Perrier de la Bâthie (1934) or subgen. Impatiens sensu Fischer and Rahelivololona (2002), as well as molecular dating and biogeographic analyses of the Malagasy *Impatiens*.
